# Incursion of Highly Pathogenic Avian Influenza A(H5N1) Clade 2.3.4.4b Virus, Brazil, 2023

**DOI:** 10.3201/eid3003.231157

**Published:** 2024-03

**Authors:** Andreina Carvalho de Araújo, Laura Morais Nascimento Silva, Andrew Yong Cho, Márcio Repenning, Deyvid Amgarten, Ana Paula de Moraes, Fernanda Malta, Michael Miller, Erick G. Dorlass, Soledad Palameta, Daniele Bruna L. Oliveira, Jansen de Araújo, Clarice Weis Arns, Edison L. Durigon, João Renato R. Pinho, Dong-Hun Lee, Helena Lage Ferreira

**Affiliations:** University of São Paulo, Pirassununga, Brazil (A.C. Araujo, L.M.N. Silva, D.B.L. Oliveira, J. De Araujo, E.L. Durigon, J.R.R. Pinho, H.L. Ferreira);; Konkuk University, Gwangjin-gu, South Korea (A.Y. Cho, D.-H. Lee); FURG, Rio Grande, Brazil (M. Repenning);; Albert Einstein Israelite Hospital, São Paulo, Brazil (D. Amgarten, F. Malta, E.G. Dorlass, D.B.L. Oliveira, J.R.R. Pinho);; UNICAMP, Campinas, Brazil (A.P. de Moraes, M. Miller, S. Palameta, C.W. Arns);; Institut Pasteur, São Paulo, Brazil (E.L. Durigon)

**Keywords:** influenza, highly pathogenic avian influenza virus, H5N1, wild bird, phylogenetic analysis, phylodynamic analysis, viruses, Brazil, respiratory infections, zoonoses

## Abstract

We report 4 highly pathogenic avian influenza A(H5N1) clade 2.3.4.4.b viruses in samples collected during June 2023 from Royal terns and Cabot’s terns in Brazil. Phylodynamic analysis revealed viral movement from Peru to Brazil, indicating a concerning spread of this clade along the Atlantic Americas migratory bird flyway.

Highly pathogenic avian influenza viruses (HPAIVs) have caused substantial economic losses in the poultry industry and potentially threaten public health. Since its first identification in 1996, H5Nx HPAIVs, Gs/GD lineage, have evolved into multiple genotypes through reassortment across decades (*1*–*3*).

In late 2020, novel reassortant clade 2.3.4.4b H5N1 HPAIVs emerged and became predominant in Europe (*1*). The first detection of clade 2.3.4.4 b H5N1 viruses in North America occurred through transatlantic spread via wild birds in late 2021 (*2*). From late 2021 to early 2022, multiple reassortant viruses have been naturally generated by recombination with North American low pathogenicity avian influenza virus (LPAIV) internal genes. In late October 2022, South America countries including Argentina, Bolivia, Brazil, Chile, Colombia, Ecuador, Paraguay, Peru, Uruguay, and Venezuela reported clade 2.3.4.4 b H5N1 HPAIV detection in domestic and wild birds (*3*,*4*). Human infections were also reported for the first time in South America (*3*,*5*). We report 4 clade 2.3.4.4b H5N1 HPAIVs sequenced from wild bird carcasses collected in Brazil in June 2023. 

In June 2023, we collected swab samples from Royal terns (*Thalasseus maximus*) and Cabot’s terns (*Thalasseus acuflavidus*) in Brazil, from which we detected and sequenced 4 H5N1 HPAIVs: A/Thalasseus_maximus/Brazil-ES/23ES1A0008/2023 (TM/BR08/23), A/Thalasseus_acuflavidus/Brazil-ES/23ES1A0009/2023 (TA/BR09/23), A/Thalasseus acuflavidus/Brazil-ES/23ES1A0025/2023 (TA/BR25/23), and A/Thalasseus maximus/Brazil-ES/23ES1A0026/2023 (TM/BR26/23) (Appendix 1). We obtained complete genome sequences for TM/BR08/23 and TM/BR09/23 and partial sequences for TA/BR25/23 and TM/BR26/23 (GISIAD [https://www.gisaid.org] accession nos. EPI_ISL_18130597, EPI_ISL_18130622, EPI_ISL_18130627, and EPI_ISL_18130628) (Appendix 1 Table 1).

All H5N1 isolates possessed polybasic amino acid sequences at the hemagglutinin (HA) cleavage site (PLREKRKKR/GLF). The isolates shared high sequence identities (99.59%–100%) across all 8 genes. BLAST search (https://blast.ncbi.nlm.nih.gov) showed all 8 genes shared high identities (99.59%–100%) to the recent H5N1 HPAIVs isolated from samples obtained in Chile and other South American countries. Using maximumplikelihood phylogenies, we noted that all internal genes (polymerase basic [PB] 2, PB1, polymerase acidic [PA], nucleoprotein [NP], matrix [M], nonstructural [NS]) clustered with the B3.2 genotype, a reassortant genotype identified in the United States in early 2022. The B3.2 genotype comprises North America–origin PB2, PB1, NP, and NS and Eurasia–origin PA, HA, NA, and M. We observed no evidence of reassortment, indicating the viruses were direct descendants of genotype B3.2 (Appendix 1 Figure 1) (*6*). Bayesian phylogeny of the HA gene revealed the H5N1 viruses from Brazil formed a well-supported cluster. We estimated the time to most recent ancestor to be May 13, 2023 (95% highest posterior density April 15, 2023–June 10, 2023), suggesting the H5N1 HPAIVs emerged ≈1 month before the detection in the carcasses of wild terns (Figure).

**Figure:**
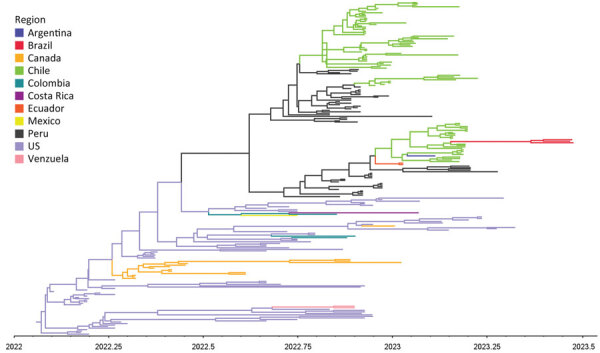
Maximum clade credibility phylogenetic tree of hemagglutinin gene based on discrete trait analysis of geographic location of wild bird carcasses identified as harboring highly pathogenic avian influenza A(H5N1) clade 2.3.4.4b virus, Brazil, 2023. The time scale is shown on the horizontal axis. Each branch is colored according to geographic region.

We estimated the incursion of genotype B3.2 into South America to be around August 14, 2022 (95% highest posterior density July 3, 2022–September 21 2022). Discrete trait analysis of geographic location suggested the source of H5N1 HPAIV was from North America, with frequently observed viral movement from Peru to Chile (Figure). The viral transition from Chile to Brazil was highly supported. However, the long branch between the two countries suggests a lack of data to be filled (Figure; Appendix 1 Tables 2, 3). Discrete trait analysis after minimizing the sampling bias showed similar results to the initial analysis (Appendix 1 Figure 2).

We evaluated mammalian molecular markers by using the H5N1 HPAIVs and the human virus from Chile (A/Chile/25945/2023) (*5*). The HA protein sequences of the H5N1 HPAIVs had amino acids related to those with a binding affinity to avian-like (α-2,3 sialic acid) receptors (188T, 210A, 222Q, and 224G in H5 numbering) (*7*,*8*). The HA protein sequences had 3 minor substitutions associated with increased binding affinity of the HA receptor to a human-like receptor (α-2,6 sialic acid) (S123P, S133A, and T156A in HA) (Appendix 1 Table 4). All isolates exhibited L89V, K389R, and V598T in PB2; N30D, I43M, and T215A in M; and P42S and ESEV PDZ binding motif mutations in NS, known to increase virulence in mice. The Chilean virus harbored more amino acid substitutions known to be associated with increased viral replication in mammals, including Q591K and D701N in PB2, A515T in PA, and L98F and I101M in NS (Appendix 1 Table 4).

The reassortment of H5Nx clade 2.3.4.4b HPAIVs, containing segments from both HPAIVs and LPAIVs, created a diverse genetic pool of H5 clade 2.3.4.4 that is continuously emerging in various countries (*9*). Novel reassortment of Eurasian clade 2.3.4.4 HPAIV with North America LPAIVs was reported in 2014–2015 and 2022–2023 (*6*,*10*). South America has been largely unaffected by the HPAIV epizootic in the past decade, but more countries are reporting HPAIV since its first detection in October 2022. Royal terns and Cabot’s terns are mainly coastal birds, staying on shore areas all year (P. Yorio et al., unpub. data, https://doi.org/10.1675/1524-4695-31.4.561). The terns are known to use the Atlantic Americas Flyway and move along the coast, which raises concern for the spread of H5N1 HPAIV in this region. The unprecedented global distribution and continuous generation of novel reassortant clade 2.3.4.4b HPAI H5Nx viruses call for heightened monitoring of HPAIV movement and reassortment to improve prevention and control policies.

Appendix 1 Additional information for study of incursion of highly pathogenic avian influenza A(H5N1) clade 2.3.4.4b virus, Brazil, 2023.

Appendix 2 Sequences from GISAID used for study of incursion of highly pathogenic avian influenza A(H5N1) clade 2.3.4.4b virus, Brazil, 2023.
