# Supplementary material for: Incursion of Highly Pathogenic Avian Influenza A(H5N1) Clade 2.3.4.4b Virus, Brazil, 2023
Source: Emerg Infect Dis. 2024 Mar;30(3):619–21. doi: 10.3201/eid3003.231157 (PMC10902554; doi:10.3201/eid3003.231157)

Article DOI: http://doi.org/10.3201/eid3003.231157

EID cannot ensure accessibility for supplementary materials supplied by authors. Readers who have difficulty accessing supplementary content should contact the authors for assistance.

Incursion of Highly Pathogenic Avian Influenza A(H5N1) Clade 2.3.4.4b Virus, Brazil, 2023

Appendix

Methods and Materials

Ethics Statement

The authors confirm that the ethical policies of the journal, as noted on the journal's author guidelines page, have been adhered to and the appropriate ethical review committee approval has been received. All applicable international, national, and/or institutional guidelines for the care and use of animals were followed. Sample collections were authorized by the Brazilian Institute of Environment and Natural Renewable Resources (SISBIO/IBAMA: 64261-6), by the Ethics Committee on the Use of Animals of the University of São Paulo (CEUA/FZEA-USP: 1128141120), and National System for the Management of Genetic Heritage and Associated Traditional Knowledge (SISGEN A028CF5).

Virus Detection

Cloacal swab samples from wild birds were collected in the coastal region of Espírito Santo and close to the Estuary of the Doce River in June 2023. Eighty-one individuals belonging to 48 bird species from 23 orders were sampled. All of the animals captured were healthy, except five birds found dead in the environment without any clinical signs reported. The five carcasses belonged to two species (*Thalasseus acuflavidus and Thalasseus maximus*), *Charadriiformes* order. Total RNA was extracted using MagMax core nucleic acid purification kit (Thermofisher, California, USA) from swab samples according to the manufacturer's instructions. RNA was tested for influenza A virus matrix gene by real-time reverse transcription PCR (RT-qPCR) as previously described (*1*). Influenza A positive viruses were only detected from swab samples of

four carcasses of Royal tern (*Thallasseus maximus*) and Cabot's tern (*Thallasseus acuflavidus*). (Appendix 1 Table 1).

Full-genome sequencing and assembly

All 8 genes of each sample were amplified using the *SuperScript* IV *Reverse Transcriptase kit* (Thermo Fisher Scientific, USA) using the universal primers as previously described (*2*). Library preparation was done using the Nextera XT DNA kit (Illumina). Next generation sequencing was run on NextSeq 550 (Illumina, San Diego, California, USA) with NextSeq 500/550 Mid Output Kit v2 (300-cycles). AIV genome segment sequences were obtained by reference (segments from A/Gull/CHL/227023-3/2022(H5N1), BioProject: PRJNA323817) guided assembly using Geneious Prime software version 2023.2.1 (https://www.geneious.com). Number of reads per segment ranged from 148,373 to 497339 for TM/BR08/23, 97,425 to 305,070 for TM/BR09/23, 131996 to 832140 for TM/BR25/23, 175,542 to 474,714 for TM/BR26/23. All 8 genes from samples were uploaded in the EpiFlu database of Global Initiative on Sharing All Influenza Data (GISAID: https://www.gisaid.org), under the accession numbers: EPI_ISL_18130597 and EPI_ISL_18130622 (Complete genome was obtained for TM/BR08/23 and TM/BR09/23, respectively). EPI_ISL_18130627 and EPI_ISL_18130628 (AIV genome partially sequenced of TA/BR25/23 and TM/BR26/23, respectively) (Appendix 1 Table 1)

Phylogenetic Inference

GISAID Epiflu (https://platform.gisaid.org) database and NCBI BLAST database were used to retrieve the reference sequences for comparative phylogenetic analysis (*3*). Reference sequences were obtained based on sequence identity. ElimDupes software (https://www.hiv.lanl.gov/content/sequence/elimdupesv2/elimdupes.html) was used with sequence identity thresholds set at 99.5%-100% to subsample genomes from over-represented discrete sample location traits. Complete coding regions were aligned by using MAFFT (https://mafft.cbrc.jp/alignment/software/). We generated maximum-likelihood (ML) trees of each gene segment. We used the RAxML with general time-reversible (GTR) + gamma nucleotide substitution model and 1,000 rapid bootstrap replicates. Genotype of the virus genome were assigned using GenoFLU (https://github.com/USDA-VS/GenoFLU) (*4*). For further Bayesian discrete trait analysis, sequences were selected for optimal root-to-tip regression.

Using the combined dataset from GISAID Epiflu database and NCBI BLAST database, we performed Bayesian discrete trait analysis of 228 HA genes using BEAST v1.10.4 and applied an uncorrelated log-normal distribution relaxed clock method, the Hasegawa–Kishino–Yano + gamma nucleotide substitution model, and the Gaussian Markov random field Bayesian skyride coalescent prior. Markov Chain Monte Carlo method to sample trees and evolutionary parameters was run in quadruple 100 million chains (*5*). Four independent chains were combined for adequate sampling of the posterior distribution of trees. Convergence and effective sample size was analyzed with TRACER v1.4 (https://beast.bio.ed.ac.uk/tracer) with 10% burn-in. A maximum clade credibility (MCC) tree was generated by using TreeAnnotator v1.10.4 (http://www.phylo.org/index.php/tools/treeannotator.html) and visualized with FigTree v1.4.2 (https://tree.bio.ed.ac.uk/). Viral transmission between discrete traits was analyzed using the Bayesian stochastic search variable selection using SpreaD3 v0.9.7 software.

The Bayesian discrete trait phylogeography approach used in this study can be affected severely by under or over-represented numbers of sequences with respect to location states. The influence of sampling bias was assessed by location state randomization (*6*,*7*). The discrete states were randomly swapped throughout the Bayesian phylogeography analysis and the estimated root location state frequency of each discrete state was compared. The probability of the estimated ancestral root state of each discrete state should be similar in unbiased datasets when discrete traits are randomly swapped throughout the analysis. To minimize the effect of sampling bias, sequences were down-sampled by removing redundant sequences. The sequences from 4 over-represented discrete traits, Canada, USA, Chile, and Peru were down-sampled based on the sequence identity at 99.5-100% using the ElimDupes software and 3 discrete traits containing only single sequence (Costa Rica, Mexico, and Argentina) were excluded to ensure that every group has less than 20% of total number of sequences (Appendix 1 Table 2). The down-sampled dataset contained a total of 31 sequences.

Amino acid substitutions associated with viral adaptation in mammalian hosts

To examine amino acid changes associated with mammalian adaptation, we used HA Subtype Numbering Conversion from Bacterial and Viral Bioinformatics Resource Center (https://www.bv-brc.org/) to align HA sequences from this study into H5 numbering scheme (8). Internal gene sequences were aligned using MAFFT (http://mafft.cbrc.jp/alignment/software/).

Results

For the complete data set, the root location state frequencies of discrete states were not evenly distributed (0.01-39.01%) when discrete traits were randomly swapped throughout the analysis, indicating a strong impact of sample size in this data set (Appendix 1 Table 2). The Chile category had the highest root location state frequency (59.01%) which is most likely influenced by the large number of samples in this discrete state. To reduce this bias, the data set was additionally down-sampled to have more balanced sample sizes. For the down-sampled data set, the root location state frequency of each discrete state was more similar (7.04-21.27%) to each other than that of original dataset, indicating a low impact of sample size in this analysis. The phylogeography analysis using the down-sampled data set showed identical pattern of virus transmissions with the complete dataset (Appendix 1 Table 3). Despite uneven sample size in the complete data set, both analyses showed identical migration pattern of virus, suggesting the discrete trait analysis results of complete dataset adequately represent the overall transmission routes of virus. Additionally, Brazilian 2.3.4.4b H5N1 HPAIV sequences contain amino acid residues known to be associated with increased virulence in mammals (Appendix 1 Table 4). Our data presents the most probable transmission routes using all available data, and the results strongly indicate the virus spread from USA to Brazil through Peru and Chile.

References

1. Spackman E, Senne DA, Bulaga LL, Myers TJ, Perdue ML, Garber LP, et al. Development of real-time RT-PCR for the detection of avian influenza virus. Avian Dis. 2003;47(Suppl):1079–82. PubMed https://doi.org/10.1637/0005-2086-47.s3.1079

2. Lee DH. Complete Genome Sequencing of Influenza A Viruses Using Next-Generation Sequencing. Methods Mol Biol. 2020;2123:69–79. PubMed https://doi.org/10.1007/978-1-0716-0346-8_6

3. Shu Y, McCauley J. GISAID: Global initiative on sharing all influenza data - from vision to reality. Euro Surveill. 2017;22:30494. PubMed https://doi.org/10.2807/1560-7917.ES.2017.22.13.30494

4. Youk S, Torchetti MK, Lantz K, Lenoch JB, Killian ML, Leyson C, et al. H5N1 highly pathogenic avian influenza clade 2.3.4.4b in wild and domestic birds: Introductions into the United States and reassortments, December 2021–April 2022. Virology. 2023;587:109860.

5. Suchard MA, Weiss RE, Sinsheimer JS. Bayesian selection of continuous-time Markov chain evolutionary models. Mol Biol Evol. 2001;18:1001–13. PubMed https://doi.org/10.1093/oxfordjournals.molbev.a003872

6. Edwards Ceiridwen J, Suchard Marc A, Lemey P, Welch John J, Barnes I, Fulton Tara L, et al. Ancient Hybridization and an Irish Origin for the Modern Polar Bear Matriline. Current Biology. 2011;21(15):1251-8.

7. Hicks JT, Dimitrov KM, Afonso CL, Ramey AM, Bahl J. Global phylodynamic analysis of avian paramyxovirus-1 provides evidence of inter-host transmission and intercontinental spatial diffusion. BMC Evolutionary Biology. 2019;19(1):108.

8. Burke DF, Smith DJ. A recommended numbering scheme for influenza A HA subtypes. PLoS One. 2014;9:e112302. PubMed https://doi.org/10.1371/journal.pone.0112302

Appendix Table 1. Clade 2.3.4.4 b H5N1 high pathogenicity avian influenza virus isolated in this study.

ID	Subtype	Collection Date	Species identified	Location	RT-qPCR M gene (Ct)	GISAID ID
TM/BR08/23	H5N1	2023-06	Royal tern	Espirito Santo	Positive (28.57)	EPI_ISL_18130597
TA/BR09/23	H5N1	2023-06	Cabot's tern	Espirito Santo	Positive (22.51)	EPI_ISL_18130622
TA/BR25/23	H5N1	2023-06	Cabot's tern	Espirito Santo	Positive (20.69)	EPI_ISL_18130627
TM/BR26/23	H5N1	2023-06	Royal tern	Espirito Santo	Positive (28.67)	EPI_ISL_18130628

Appendix Table 2. Number of sequences in each discrete state and root location state frequency estimates result from location state randomization analysis.

	Number of sequences and root location state frequency (%) of each discrete state			
	Complete dataset (n=228)		Down-sampled dataset (n=31)	
Discrete state	Number of sequences (%)	Root location state frequency	Number of sequences (%)	Root location state frequency (%)
Chile	77 (33.8%)	59.03%	5 (16.1%)	13.58%
USA	72 (31.6%)	39.01%	6 (19.4%)	14.10%
Peru	51 (22.4%)	0.01%	6 (19.4%)	18.47%
Canada	15 (6.6%)	1.32%	4 (12.9%)	21.27%
Brazil	3 (1.3%)	0.19%	3 (9.7%)	7.04%
Colombia	3 (1.3%)	0.21%	3 (9.7%)	11.00%
Venezuela	2 (0.9%)	0.03%	2 (6.5%)	7.18%
Ecuador	2 (0.9%)	0.04%	2 (6.5%)	7.36%
Argentina	1 (0.4%)	0.01%	-	-
Costa Rica	1 (0.4%)	0.07%	-	-
Mexico	1 (0.4%)	0.08%	-	-

Appendix Table 3. 2.3.4.4b H5N1 HPAIV transmission matrix of Bayesian stochastic search variable selection analysis of geographic location

Dataset	From	To	Bayes factor	Posterior Probability
Complete (n=228)	USA	Canada	30387.8	1.00
	Peru	Chile	18566.7	1.00
	USA	Columbia	4700.1	1.00
	Chile	Brazil	194.1	0.95
	Peru	Ecuador	119.3	0.93
	Chile	Argentina	87.6	0.90
	USA	Venezuela	51.0	0.85
	USA	Peru	41.7	0.82
	Colombia	Costa Rica	13.2	0.59
	Colombia	Mexico	9.5	0.50
Down-sampled (n=31)	Peru	Chile	1196.3	0.99
	Chile	Brazil	126.4	0.95
	USA	Columbia	98.4	0.94
	Peru	Ecuador	28.9	0.82
	USA	Canada	12.0	0.66
	USA	Venezuela	11.6	0.65
	USA	Peru	5.9	0.50

Appendix Table 4. Amino acid substitutions of Brazilian 2.3.4.4b H5N1 HPAIV, June 2023. Sequences with amino acid residues known to be associated with increased virulence in mammals were highlighted in pink.

	HA (H5 numbering) *									PB2†					PA‡	NP[#]		M1§			NS**				
Isolates	D94N	S123P	S133A	N154D	T156A	T188I	V210I	Q222L	G224S	L89V	D256G	Q591K	E627K	D701N	A515T	Y52NQH	F313Y	N30D	I43M	T215A	P42S	80-84 deletion	L98F	I101M	ESEV
Brazil isolates	S	P	A	N	A	T	A	Q	G	V	D	Q	E	D	D	Y	F	D	M	A	S	TIASV	M	D	ESEV
A/Chile/25945/2023	S	P	A	N	A	T	A	Q	G	V	D	K	E	N	T	Y	F	D	M	A	S	TIASV	F	M	ESEV

* D94N, S123P, S133A, N154DN154D, T156A, T188I, V210I, Q222L, and G224S mutations in HA are known to be associated with increased binding to human-like receptor (α-2,6 sialic acid).
† L89V and D256G mutations in PB2 are known to be associated with increased virulence in mice. Q591K, E627K, and D701N mutations in PB2 are known to be associated with increased viral replication in mammals.
‡ A515T mutation in PA is known to be associated with H5 transmissibility in ferrets.
# Y52NQH and F313Y is known to be associated with human BTN3A3 evasion
§ N30D, I43M, T215A mutations in M1 are known to be associated with increased virulence in mice.
** P42S, 80-84 deletion, and ESEV PDZ binding motif mutations in NS are known to be associated with increased virulence in mice.

Appendix Figure 1 (see following pages). Maximum-likelihood phylogenetic tree using eight genes of H5N1 HPAIV: A) PB2, B) PB1, C) PA, D) HA, E) NP, F) NA, G) MP, and H) NS. Node values represent bootstrap values. Tips of Brazilian isolates are labeled in red. Branches of South American isolates are highlighted in yellow shade. Scale bar indicates nucleotide substitutions per site.

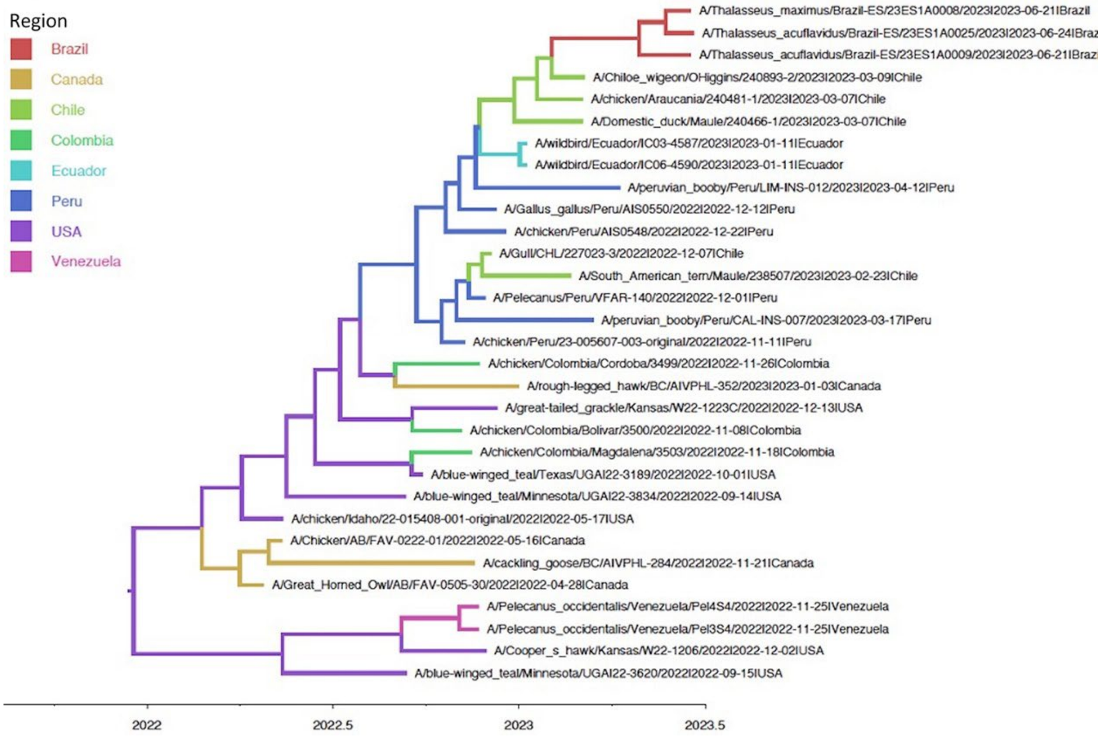

Appendix Figure 2. Bayesian phylogenetic maximum clade credibility (MCC) tree using the down-sampled data set of the clade 2.3.4.4b H5N1 HPAIVs. Branches are colored according to discrete traits of geographic regions. The scale axis indicates the branch length by year.

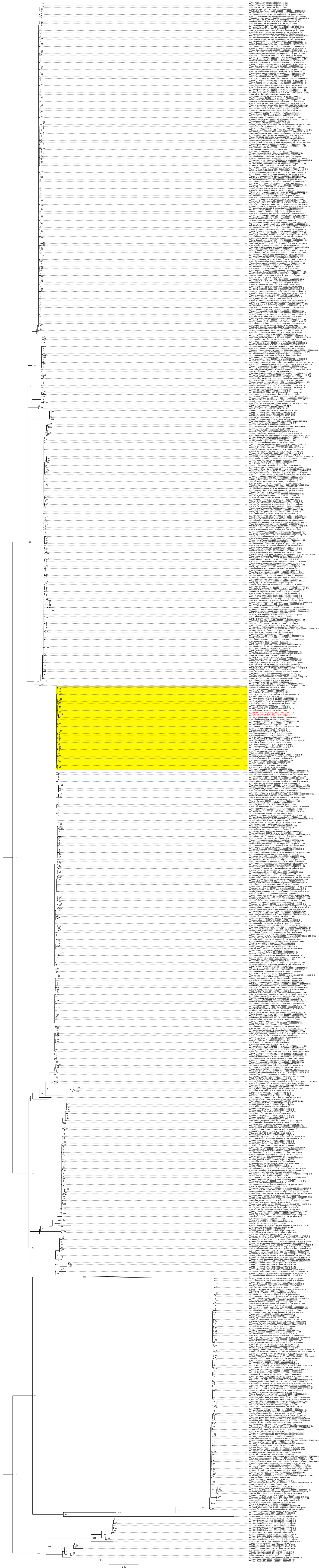
A

B

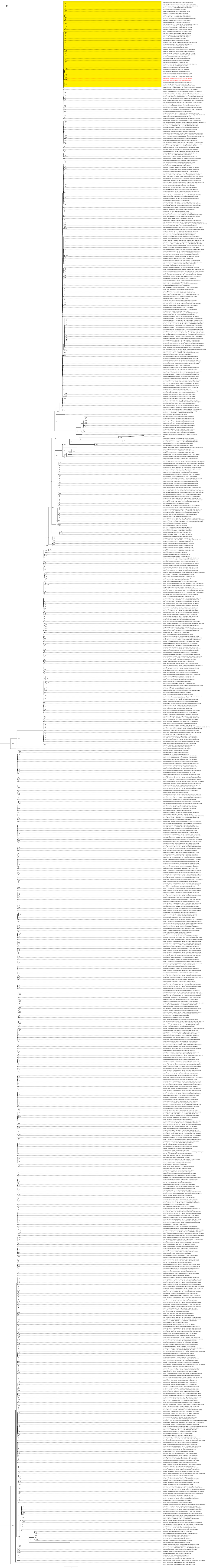

C

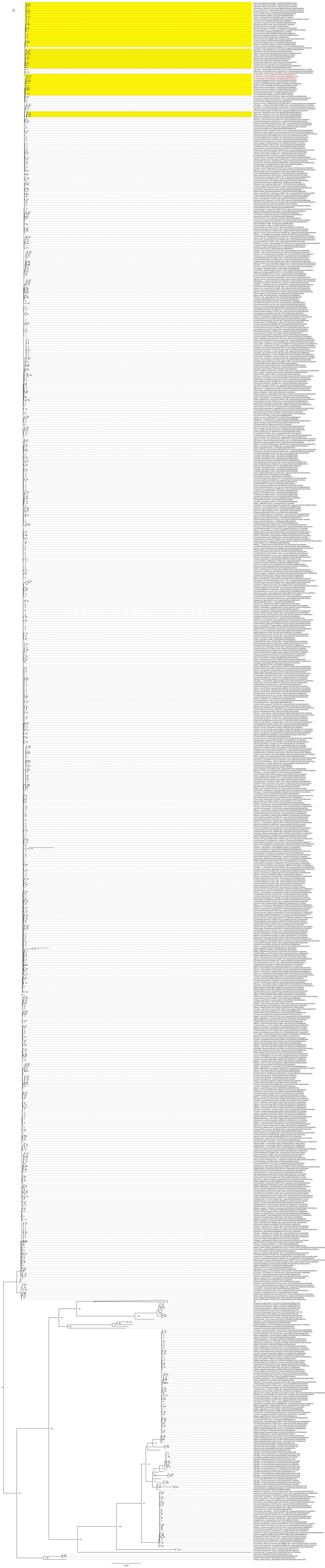

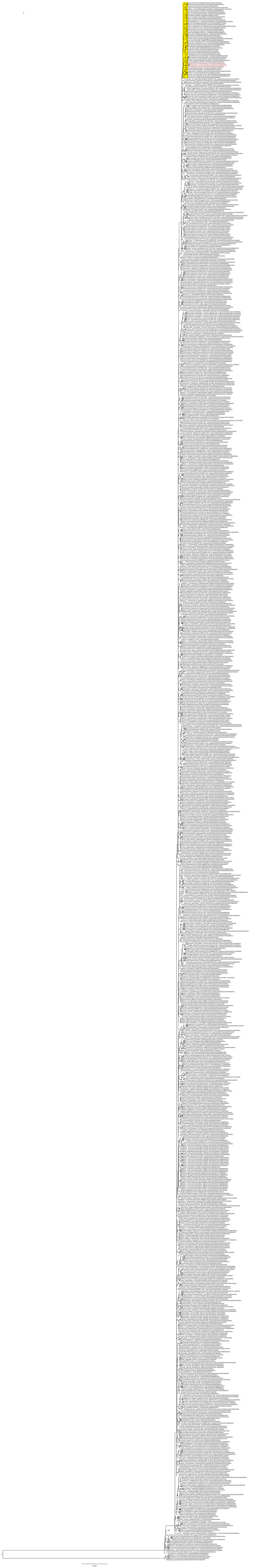

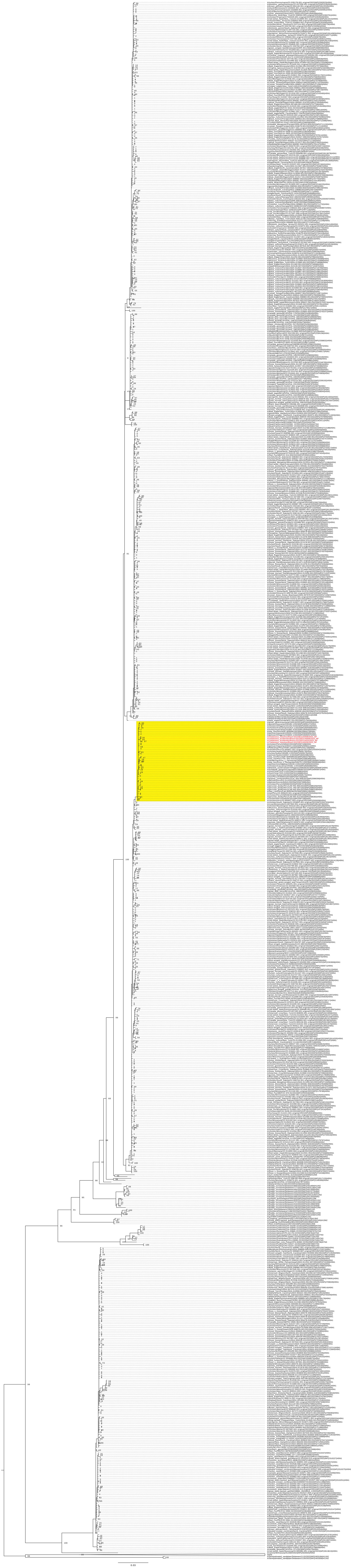
0.03

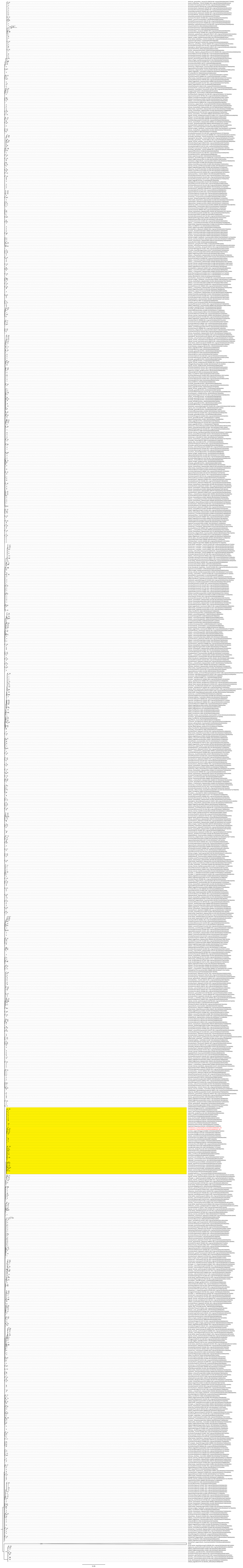

A/Snowy_Owl/New_York/USDA-008969-001/2022|NA|2703190|H5N1
A/Great_Horned_Owl/Wyoming/USDA-013016-002/2022|NA|2712798|H5N1
A/Snow_Goose/North_Dakota/USDA-010663-008/2022|NA|2702697|H5N1
A/Snow_Goose/South_Dakota/USDA-009478-002/2022|NA|2703089|H5N1
A/chicken/Minnesota/22-014503-002-original/2022|NA|2258886|H5N1
A/Red-tailed_Hawk/Wisconsin/USDA-010924-001/2022|NA|2703228|H5N1
A/turkey/Minnesota/22-012287-001-original/2022|NA|2246047|H5N1
A/goose/Missouri/USDA-012338-029/2022|NA|2712751|H5N1
A/RT-Hawk/ON/FAV-0210/2022|NA|2247005|H5N1
A/Red-tailed_Hawk/South_Dakota/USDA-009041-001/2022|NA|2703324|H5N1
A/turkey/South_Dakota/22-010639-001-original/2022|NA|2179243|H5N1
A/goose/Missouri/USDA-012338-026/2022|NA|2712775|H5N1
A/Red-tailed_Hawk/Minnesota/USDA-011871-013/2022|NA|2703880|H5N1
A/Eagle/Missouri/USDA-012338-007/2022|NA|2712696|H5N1
A/Great_Horned_Owl/Minnesota/USDA-012760-003/2022|NA|2712176|H5N1
A/goose/Ohio/USDA-008995-002/2022|NA|2712389|H5N1
A/Chicken/BC/FAV-0460-OS/2022|NA|2056423|H5N1
A/turkey/BC/AIVPHL-91/2022|NA|2428101|H5N1
A/chicken/Idaho/22-015407-001-original/2022|NA|2259358|H5N1
A/chicken/Idaho/22-011347-004-original/2022|NA|2181822|H5N1
A/chicken/Idaho/22-015657-001-original/2022|NA|2259446|H5N1
A/chicken/Idaho/22-015655-001-original/2022|NA|2259430|H5N1
A/chicken/Idaho/22-014175-003-original/2022|NA|2258646|H5N1
A/goose/Wyoming/USDA-012665-003/2022|NA|2711783|H5N1
A/chicken/Wyoming/22-009326-002-original/2022|NA|2035152|H5N1
A/chicken/Colorado/22-011826-002-original/2022|NA|2243762|H5N1
A/raccoon/Washington/22-018406-002-original/2022|NA|2182118|H5N1
A/guinea_fowl/Illinois/22-007382-001-original/2022|NA|2014028|H5N1
A/Snow_Goose/North_Dakota/USDA-009481-008/2022|NA|2702529|H5N1
A/Snow_Goose/North_Dakota/USDA-011039-005/2022|NA|2703622|H5N1
A/Red_Fox/MB/FAV-370-01/2022|NA|2235554|H5N1
A/Red-tailed_Hawk/Michigan/USDA-012854-001/2022|NA|2703292|H5N1
A/Green-winged_Teal/North_Carolina/USDA-001673-001/2022|NA|2712726|H5N1
A/Red_Fox/ON/FAV-0605-01/2022|NA|2235602|H5N1
A/Snow_Goose/North_Dakota/USDA-010663-013/2022|NA|2702600|H5N1
A/black_vulture/North_Carolina/W22-1213/2022|NA|2510362|H5N1
A/chicken/Virginia/22-033680-002-original/2022|NA|2263785|H5N1
A/duck/New_Jersey/22-015259-001-original/2022|NA|2259246|H5N1
A/Black_Vulture/Florida/USDA-008154-001/2022|NA|2708622|H5N1
A/Bald_Eagle/North_Carolina/USDA-007917-003/2022|NA|2707955|H5N1
A/Skunk/AB/FAV-0416-02/2022|NA|2236762|H5N1
A/chicken/Michigan/22-012855-001-original/2022|NA|2246231|H5N1
A/Bald_Eagle/Missouri/USDA-012338-021/2022|NA|2707595|H5N1
A/Ross_s_Goose/North_Dakota/USDA-009481-021/2022|NA|2705266|H5N1
A/Snow_Goose/Minnesota/USDA-010305-001/2022|NA|2702296|H5N1
A/American_Crow/North_Dakota/USDA-011564-002/2022|NA|2709286|H5N1
A/chicken/Minnesota/22-010928-001-original/2022|NA|2179579|H5N1
A/turkey/South_Dakota/22-008242-001-original/2022|NA|2016934|H5N1
A/fox/Michigan/22-014536-007-original/2022|NA|2258910|H5N1
A/Bald_Eagle/Minnesota/USDA-011695-001/2022|NA|2707246|H5N1
A/American_Wigeon/North_Carolina/USDA-001672-009/2022|NA|2709205|H5N1
A/Red_fox/ON/FAV-0301-05/2022|NA|2259581|H5N1
A/Bald_Eagle/Wisconsin/USDA-012897-001/2022|NA|2708707|H5N1
A/American_wigeon/North_Carolina/AH0182517/2022|NA|1985929|H5N1
Swine
A/red-tailed_hawk/Wyoming/23-005384-001-original/2023|NA|2613621|H5N1
A/glaucous_gull/California/23-010132-001-original/2023|NA|2614149|H5N1
A/Bald_Eagle/Alaska/USDA-013831-001/2022|NA|2708513|H5N1
A/Bald_Eagle/Alaska/USDA-013001-001/2022|NA|2708545|H5N1
A/trumpeter_swan/BC/AIVPHL-364/2023|NA|2429284|H5N1
A/chicken/California/22-035144-001-original/2022|NA|2263497|H5N1
A/western_gull/California/23-011273-001-original/2023|NA|2614221|H5N1
A/turkey_vulture/California/23-016937-001-original/2023|NA|2614613|H5N1
A/turkey_vulture/California/23-011452-002-original/2023|NA|2614269|H5N1
A/red_fox/Alaska/22-016300-001-original/2022|NA|2473505|H5N1
A/Bald_Eagle/BC/OTH-33-36/2022_H5N1|NA|2219868|H5N1
0.05

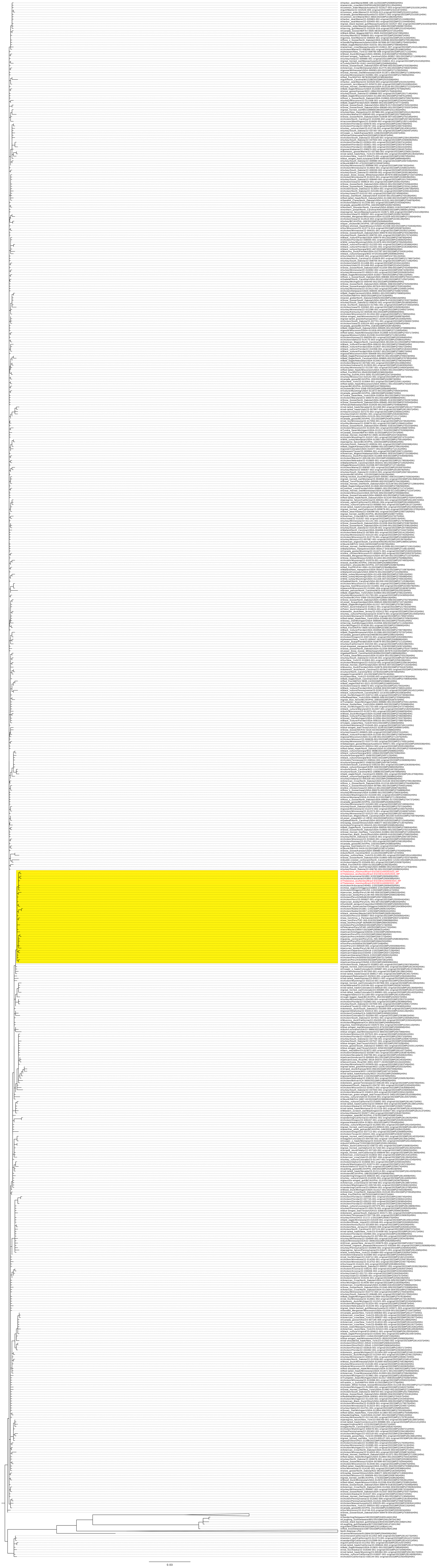
0.03

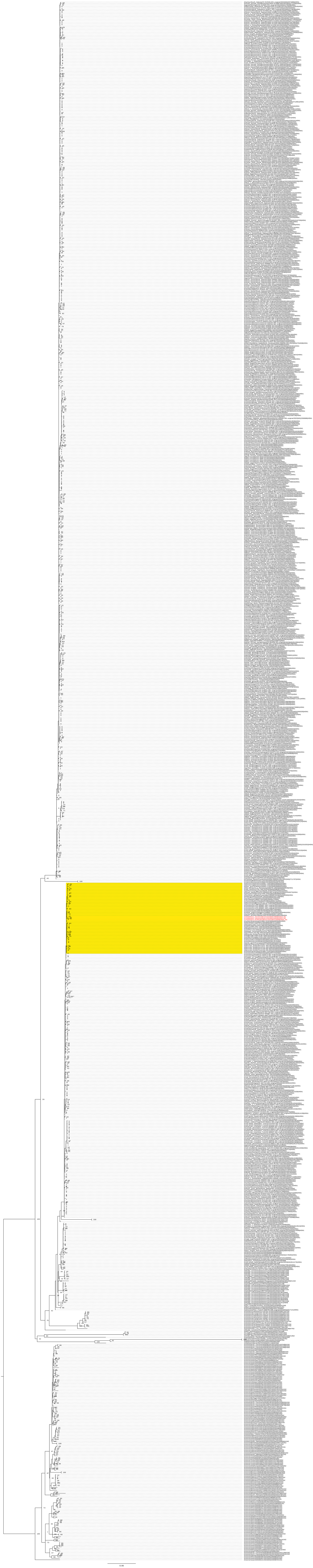

Supplement: Appendix 1 — Additional information for study of incursion of highly pathogenic avian influenza A(H5N1) clade 2.3.4.4b virus, Brazil, 2023. [file 23-1157-Techapp-s1.pdf]
